# Can a new role, the (Trainee) Associate Psychological Practitioner (T/APP), add value in General Practice? Results from the pilot year evaluation

**DOI:** 10.1017/S1463423622000482

**Published:** 2022-09-29

**Authors:** Miranda Budd, Rebecca Gardner, Gita Bhutani, Kathryn Gardner, Ameera Iqbal, Charlotte Harding, Clare Baguley, Umesh Chauhan

**Affiliations:** 1 Consultant Clinical Psychologist, Lancashire and South Cumbria NHS Foundation Trust (LSCft), Preston, Lancashire, UK; 2 Assistant Psychologist (LSCft), Preston, Lancashire, UK; 3 Director of Psychological Professions (LSCft), Preston, Lancashire, UK; 4 Senior Lecturer in Psychology (UCLan), Preston, Lancashire, UK; 5 Trainee Associate Psychological Practitioner (LSCft), Preston, Lancashire, UK; 6 HEE NW Clinical Workforce Lead (Health Education England, North West), Manchester, UK; 7 GP and Professor of Primary Care (UCLan and Pendle View Medical Centre), Preston, Lancashire, UK

**Keywords:** APP, ARRs, General Practice, mental health, prevention, promotion, psychology graduate, TAPP

## Abstract

**Background:**

The deployment of (Trainee) Associate Psychological Practitioners (T/APPs) to deliver brief psychological interventions focusing on preventing mental health deterioration and promoting emotional wellbeing in General Practice settings is a novel development in the North West of England. As the need and demand for psychological practitioners increases, new workforce supply routes are required to meet this growth.

**Aims:**

To evaluate the clinical impact and efficacy of the mental health prevention and promotion service, provided by the T/APPs and the acceptability of the role from the perspective of the workforce and the role to T/APPs, patients and services.

**Methods:**

A mixed-methods design was used. To evaluate clinical outcomes, patients completed measures of wellbeing (WEMWBS), depression (PHQ-9), anxiety (GAD-7) and resilience (BRS) at the first session, final session and at a 4–6 week follow-up. Paired-samples t-tests were conducted comparing scores from session 1 and session 4, and session 1 and follow-up for each of the four outcome measures. To evaluate acceptability, questionnaires were sent to General Practice staff, T/APPs and patients to gather qualitative and quantitative feedback on their views of the T/APP role. Quantitative responses were collated and summarised. Qualitative responses were analysed using inductive summative content analysis to identify themes.

**Results:**

T-test analysis revealed clinically and statistically significant reductions in depression and anxiety and elevations in wellbeing and resiliency between session 1 and session 4, and at follow-up. Moderate–large effect sizes were recorded. Acceptability of the T/APP role was established across General Practice staff, T/APPs and patients. Content analysis revealed two main themes: positive feedback and constructive feedback. Positive sub-themes included accessibility of support, type of support, patient benefit and primary care network benefit. Constructive sub-themes included integration of the role and limitations to the support.

**Conclusions:**

The introduction of T/APPs into General Practice settings to deliver brief mental health prevention and promotion interventions is both clinically effective and acceptable to patients, General Practice staff and psychology graduates.

## Introduction

The NHS Long-Term Plan (LTP) (NHS England, 2019) highlights the importance of care in the community and increasing provision of and access to psychological interventions. It recognises the importance of creating thriving, healthy communities with sustainable health and care provision, which is dependent on developing new ways of working in partnership with people and communities. Primary care networks (PCNs) form a key building block of the LTP. Bringing practices together can improve the ability to recruit and retain staff and to provide a wider range of services to patients. In Lancashire and South Cumbria, there are 41 PCNs, with the largest having a patient list of over 90 000 individuals. PCNs are expected to think about the wider health of their population and take a proactive approach to identifying and managing population health, dependent on local need (Baird and Beech, 2020).

Mental health is a core part of the business in primary care. It is estimated that one in three GP appointments involves a mental health component (London Strategic Clinical Network for Mental Health, 2014). In a survey of 1000 GPs published in 2018, 66% reported that the proportion of patients needing help with their mental health had increased over the previous 12 months (Mind, 2018). This is pre-pandemic, following COVID-19, demand is increasing (Molodynski *et al.*, 2021) in line with increasing mental health difficulties in the community (Jia *et al.*, 2020). Yet, with GP appointment slots providing approximately 10 minutes per individual, being able to meet mental health need within that time frame feels like an insurmountable problem. Perhaps the time pressure is one reason antidepressant medication prescriptions are increasing (Naylor *et al.*, 2012).

### The case for mental health prevention and promotion

With so many individuals presenting in General Practice with mental health need, a proactive approach as opposed to a reactive one is required (Carbone, 2020). There is a wealth of organisational and government guidelines available that make a clear case for the prevention of mental ill health and promotion of emotional wellbeing in a General Practice settings (see Budd *et al.*, 2021 for a review). The Royal College of General Practitioners (RCGP) set out 12 recommendations for mental health promotion and prevention within UK General Practice (Thomas *et al.*, 2016). They emphasise that focussing on mental health prevention will help to reduce illness, save lives, save money, reduce General Practice workload and promote resilience and good mental health. In 2020, Public Health England sought to place mental health prevention on an equal footing to programmes that seek to reduce smoking and rates of obesity in their planning resource ‘The Prevention Concordant for Better Mental Health’ (2020). It has been predicted that by 2026, the UK will not be able to cope with the cost of mental health need (Knapp and McDaid, 2011), an estimate that was made pre-COVID-19. The same authors used economic modelling to demonstrate that promotion and prevention strategies could be a clear solution due to their value for money over the long term. The framework for mental health research (Department for Health and Social Care, 2017) highlights a concern that despite the urgency and scale of the challenge, mental health research is lagging behind many other areas, meaning that improvements in prevention is progressing too slowly.

### Psychologists working in PCNs

The provision of mental health care in primary care in countries outside of the UK is variable and little information is available. In Western European countries, specialist mental health staff members are included in primary care teams in only England and Finland (WHO, 2008). In Australia, in recent years, there have been two primary mental health care reforms, the ‘Access to Allied Psychological Services’ (ATAPS) project (2001) and the ‘Better Outcomes in Mental Health Care’ initiative (2006) promoted the integration of medical and psychological care. A review of the initiatives found good uptake, which the authors concluded meant the programmes were addressing unmet need (Bassilios *et al.*, 2010). Lockhart (2006), however, described issues with collaboration and problematic referral practices between the GPs and mental health workers.

Progression towards community mental health care in most African countries is reportedly hindered by a lack of resources (Alem *et al.*, 2008). There is general agreement that mental health services should be integrated into primary health care, the lack of appropriate supervision and continuing education for primary care workers has been cited as problematic (Alem *et al.,* 2008).

Whilst in England, the General Practice Forward View (NHS England, 2016), the RCGP (Thomas, *et al.*, 2016) and Mind (2018) recommend that a wider range of practice staff within primary care could support mental health prevention and promotion.

In order to realise the aims of the LTP, PCNs have numerous funding streams. With expanding the workforce being a top priority for primary care, one funding stream is the ‘Additional Roles Reimbursement Scheme payments’, which is reimbursement of the salary for new roles being recruited into General Practice, along with certain on costs. The role ‘Mental Health Practitioner’ (MHP) has been added to the scheme in 2021/22. MHP is an umbrella term for a number of qualified professionals who are able to provide mental health care (Baird and Beech, 2020; British Medical Journal, 2021).

The demand for MHPs in General Practice settings is clear, yet there are workforce supply issues that can make recruiting and filling such roles challenging. The strategic workforce programmes across the North West Coast had anticipated difficulties in recruiting to meet the predicted demand. In 2019, ‘Closing the Gap’ (Beech *et al.*, 2019) was published, a report which highlighted NHS health care workforce shortages and key areas for action. Serious staffing shortages were reported for mental health services, with nursing roles significantly affected. In 2017, to start to address the decline in staff working in mental health services, Health Education England (HEE) unveiled a training plan. This included a focus upon a wider workforce, incorporating new roles in psychology and psychological therapies (Health Education England, 2017). Yet, despite the significant demand for psychological practitioners, traditional routes of supply into NHS roles will not meet this demand. Each year, thousands of psychology graduates complete their undergraduate degree and are keen to find employment in the field of psychological health. An ‘academic’ psychology undergraduate degree may be considered an outlier relative to other degrees which include vocational training and skill an individual for a career in clinical facing roles within the NHS. Other courses, such as medicine, speech and language therapy, occupational therapy, physiotherapy and nursing all therefore develop the necessary competencies to allow graduates entry into the workplace in a defined and structured manner. Psychology conversely involves completing a competitive three-year doctoral training degree which creates a bottleneck in terms of supply (of good candidates) and demand (the need for clinical psychologists).

With evidence then, of both demand and supply, there is a clear rationale to support an attempt to connect the two for expansion of the psychological workforce. In partnership with HEE, the University of Central Lancashire, The Innovation Agency North West Coast, Health and Wellbeing Boards across the North West Coast, The Lancashire and South Cumbria Integrated Care Board (ICB) and The Psychological Professions Network North West (PPNNW), the Postgraduate Diploma for Applied Psychological Practitioners (PGDip APP) was developed as a new educational approach and career route for psychology graduates to enter into the NHS. (Trainee) Associate Psychological Practitioners (T/APPs) work at Band 4 for 12 months, progressing to Band 5 upon qualification as an APP and remaining employed by the NHS.

This paper summarises the findings of a service evaluation of T/APPs working in NHS General Practice to deliver brief psychological interventions focusing on preventing mental health deterioration and promoting the importance of caring for your emotional wellbeing.

There were two main aims of the service evaluation. Firstly, to understand the clinical impact and efficacy of the mental health prevention and promotion service, measured in terms of patient scores on resilience, depression, anxiety and wellbeing before and after four intervention sessions with a T/APP. Second, to understand the acceptability of the workforce and the role to T/APPs, patients and the service. The overall objective of this service evaluation was to understand how the T/APPs add value to mental health care in General Practice settings.

## Method

### Service setting

The mental health prevention and promotion service was delivered across 23 PCNs in Lancashire and South Cumbria by 23 T/APPs. Within some areas, the T/APPs delivered the service across all practices within the PCN, elsewhere, T/APPs were located within one practice. This decision was made locally.

### Patients

The service offer aimed to mirror General Practice, meaning that it was open to all ages. Referrals were assessed and then accepted or signposted as appropriate including consideration of need, risk and/or urgency.

#### Inclusion criteria

1. Individuals had to be registered at a practice within the PCN.
2. Anyone who would benefit from mental health promotional or preventative advice delivered within the context of a brief intervention, determined by a General Practice staff member. Examples include patients presenting with stress, reduced wellbeing and/or common mental health symptoms such as depression or anxiety.

#### Exclusion criteria

The T/APPs offer a brief psychological intervention. The service was not meant to replicate IAPT (Improving Access to Psychological Therapies) services in General Practice settings.

1. Those who are already supported by a mental health service/engaged in therapy elsewhere.
2. Those who have a formal diagnosis of a severe mental health difficulty, where they continue to struggle and it would not be possible to adequately meet need within four sessions.
3. Those who require support from crisis services.
4. Those who have drug and alcohol misuse difficulties where their needs would be better met by the local drug and alcohol service.

### Service delivered

The T/APPs offered four × 45 min appointments, typically one per week, with a follow-up 45-minute appointment 4–6 weeks later. Appointments were delivered face to face, on the phone, or virtually, depending on the patient’s preference. During the first session, a psychological assessment was completed. The second focused upon a structured psychological formulation. Psychological work and advice given can occur during the first and second session, but it is the main focus for the third and fourth session. The T/APPs have worked from a menu-based approach, drawing upon various psychological models, depending upon presenting need. The aim of the sessions is to teach skills to people about how to look after their emotional wellbeing.

**Figure 1.**
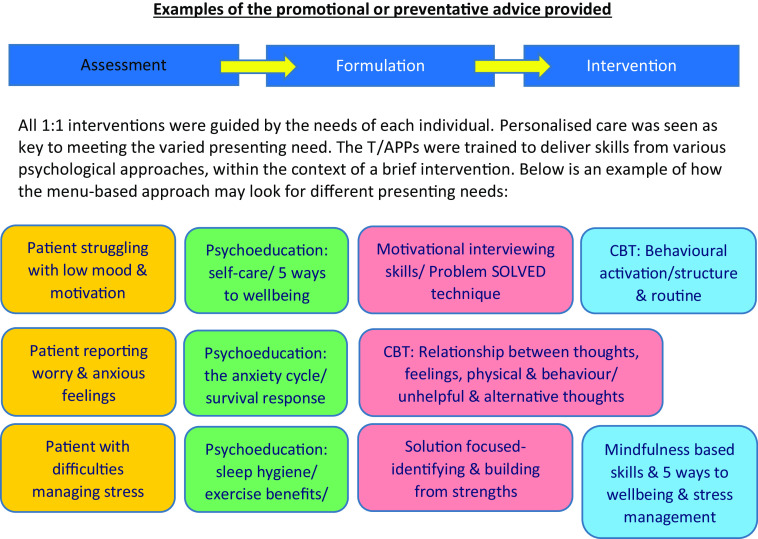
Examples of the promotional or preventative advice given.

What was offered in the individual sessions varied, dependent upon presenting need. For example, it may have been an extension of sleep hygiene advice that was started during a brief GP appointment, or more detailed self-help information about looking after emotional wellbeing. The individual sessions were informed by cognitive-behavioural theory, solution-focused theory, motivational interviewing, health coaching, compassionate mind principles, distress tolerance and mindfulness-based skills and systems theory. Figure 1 provides further examples of the promotional or preventative advice given to give context to the work conducted.

**Figure 2.**
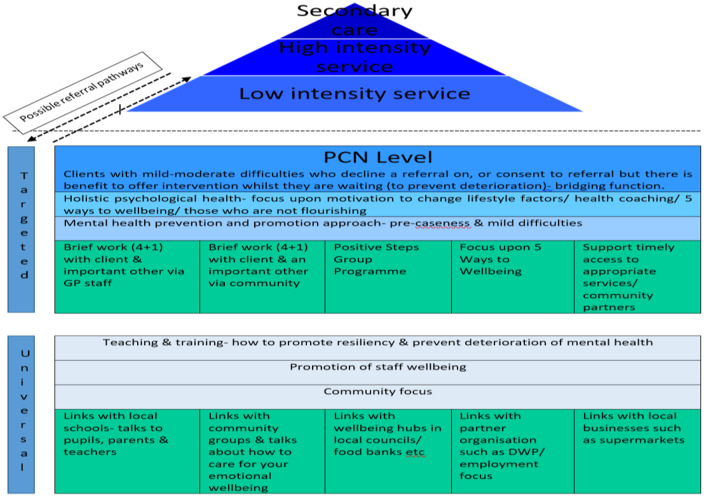
T/APP service delivery model.

Figure 2 outlines the service delivery model the T/APPs work was guided by. Three days were spent in clinical settings (‘targeted’ approach), one day working within community settings (‘universal’ approach) and one day was allocated for study and university course attendance. Additional detail about the community day, including measured impact, is beyond the scope of the current paper, so it is not included here. To support this workforce, the T/APPs received weekly 1:1 clinical supervision for an hour and weekly group supervision for an hour, both facilitated by an accredited psychological professional.

### Design overview

A mixed-methods approach was used. Quantitative and qualitative results were triangulated to capture different and multiple perspectives and address the objective of this paper, to understand how the T/APPs add value to mental health care in General Practice settings. To understand the clinical impact and efficacy of the support offered by the T/APPs (Aim 1), quantitative (patient outcomes and descriptive statistics) methods were used. This was supported by qualitative (patient, workforce and colleague experience questionnaires) methods. To understand the acceptability of the T/APP workforce (Aim 2), qualitative (patient, workforce and colleague experience questionnaires) methods were employed. This work was a service evaluation; hence, there was no control group.

### Outcome measures

Four quantitative routine outcome measures were used to assess efficacy: The Warwick and Edinburgh Mental Wellbeing Scale (WEMWBS) to measure general wellbeing (Tennant *et al.*, 2007); The Brief Resilience Scale (BRS) (Smith *et al.*, 2008) to measure resiliency; The Patient Health Questionairre-9 (PHQ-9) to measure symptoms of depression (Kroenke *et al.*, 2001) and The Generalised Anxiety Disorder-7 Scale (GAD-7) to measure symptoms of anxiety (Spitzer *et al.*, 2006). These measures were completed during the first, fourth and follow-up session (4–6 weeks after the last session).

All measures have been shown to have robust psychometric properties across multiple studies and are valid for use in primary care samples (e.g., Kroenke *et al.*, 2001; Spitzer *et al.*, 2006; Tennant et al, 2007; Cameron *et al.*, 2008; Smith *et al.*, 2008; Mavali et al, 2020).

At the end of the pilot year, an online, anonymous questionnaire link was sent to 42 General Practice staff. One or two staff members (with their consent) were selected by each T/APP to participate. This allowed for feedback to be collected from a range of PCNs, whilst not adding too much additional demand on health care staff. The questionnaire contained one quantitative and four open-ended questions and asked the respondent to identify what they thought worked well, areas for improvement and what they would like to change about the T/APP role. An online, anonymous questionnaire was also sent to all 23 T/APPs, and this contained 10 quantitative and 11 open-ended questions. The questionnaire included questions on the acceptability, demand, implementation, practicality and integration of the T/APP role, taken from Bowden *et al.*’s (2009) suggested areas of feasibility. Finally, all patients were asked to complete a ‘Patient Experience Questionnaire’ (PEQ) at discharge, containing seven open-ended questions. The respondent was asked about how they found the T/APP service overall, what had been most helpful and what had been least helpful.

## Results

### Aim 1: efficacy of the mental health prevention and promotion service

#### Referral information

Table 1 depicts the demographics of the individuals who engaged with the service.

**Table 1.** Gender, age and ethnicity breakdown for clients who received one or more wellbeing sessions with a T/APP

Demographics		Received support	
		*n* = 1209	Percentage
Gender	Male	336	27.8
	Female	871	72.0
	Non-binary	1	0.1
	Missing	1	0.1
Age (years)	0–18	116	9.6
	19–25	141	11.7
	26–35	204	16.9
	36–45	184	15.2
	46–55	202	16.7
	56–65	150	12.4
	66–75	124	10.3
	76+	88	7.3
Ethnicity	White British	1077	89.1
	White other	23	1.9
	White Irish	5	0.4
	White traveller	2	0.2
	Pakistani	61	5.0
	Indian	9	0.7
	Chinese	1	0.1
	Asian other	0	0
	Black Caribbean	10	0.8
	Black other	6	0.5
	Mixed British	2	0.2
	Mixed White/Asian	1	0.1
	Mixed White/Black	3	0.2
	African	2	0.2
	Mixed other	5	0.4
	Missing	2	0.2

**Figure 3.**
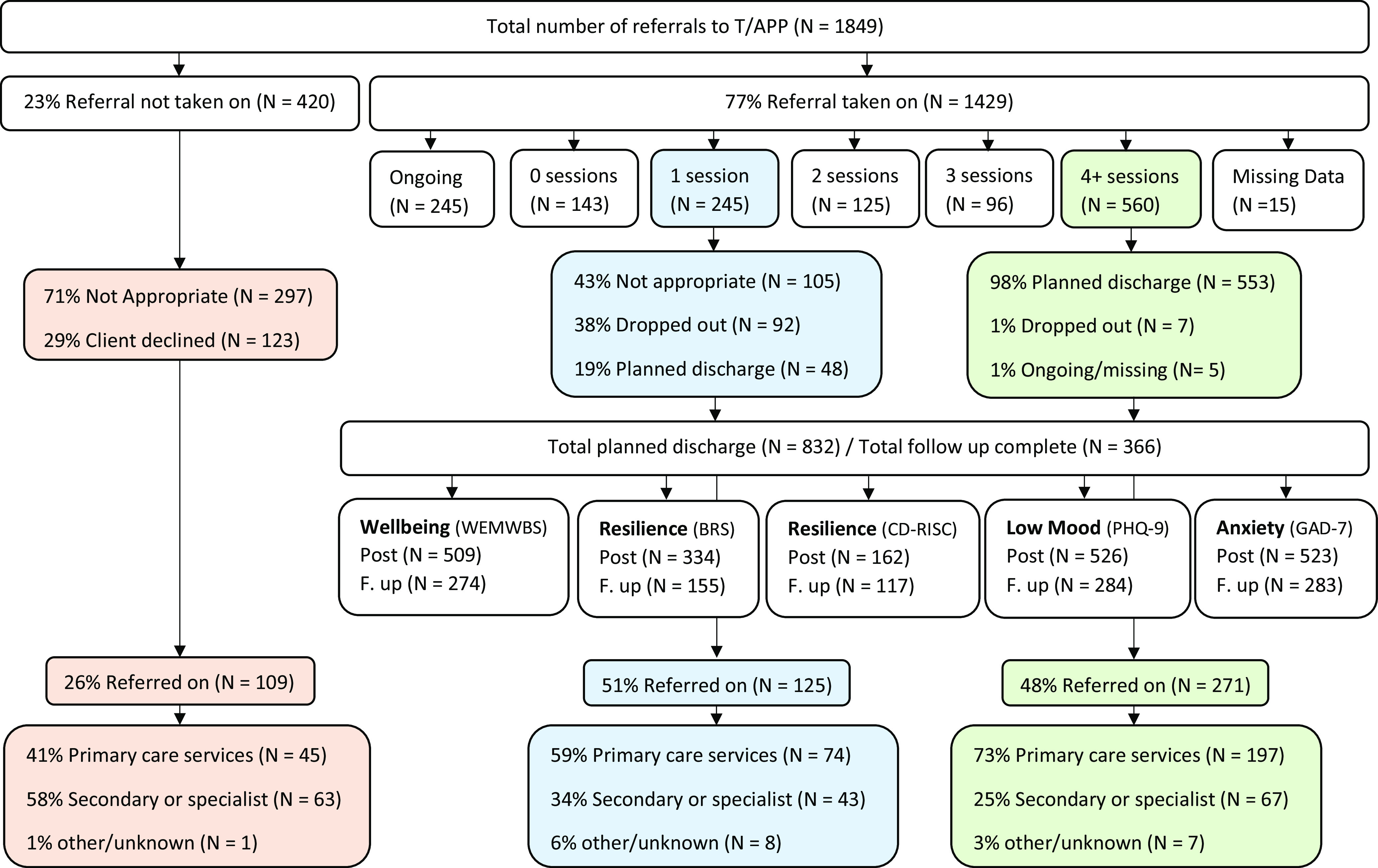
Flow chart depicting the outcomes of each clients who was referred for wellbeing support with a T/APP.

In total, 1849 people were referred for wellbeing support with a T/APP. Figure 3 depicts the care pathway for these referrals. The majority of individuals received four wellbeing sessions. Second to this, many individuals received only one session (the assessment), which allowed 51% to be referred on to a more appropriate service. Where clients declined input from the T/APP, alternative sources of support were offered. Most appointments (71%) were delivered face to face, although other mediums were also used (e.g., phone and virtual). Fifty-five per cent of individuals seen commented that COVID-19 had had a negative impact upon their mental wellbeing.

#### Clinical impact of T/APPs on patient outcomes

For each outcome measure, there were clinical improvements in wellbeing from session 1 to session 4 and at follow-up. Eight two-tailed paired samples t-test were completed to compare scores at session 1 and session 4, and at session 1 and follow-up, for each of the four outcome measures. The sample size for each analysis can be found in Figure 3, and the results are reported in Table 2.

**Table 2.** Mean, standard deviation and paired samples t-tests for the patient outcome measures comparing scores at session 1, 4 and follow-up

		Mean	*SD*	*df*	*t*	*P*	*d*
Depression (PHQ-9)	Session 1	14.09	5.48	525	24.60	<.001	1.00
	Session 4	8.60	5.46				
	Session 1	14.10	5.43	283	19.93	<.001	1.21
	Follow-up	7.39	5.68				
Anxiety (GAD-7)	Session 1	13.27	5.07	522	25.29	<.001	1.09
	Session 4	7.72	5.08				
	Session 1	13.41	4.93	282	20.60	<.001	1.29
	Follow-up	6.75	5.36				
Wellbeing (WEMWBS)	Session 1	35.97	9.11	508	−22.38	<.001	−0.96
	Session 4	45.32	10.32				
	Session 1	36.37	9.36	273	−17.68	<.001	−1.13
	Follow-up	47.93	11.05				
Resilience (BRS)	Session 1	2.62	0.72	333	−13.24	<.001	−.62
	Session 4	3.07	0.73				
	Session 1	2.64	0.76	154	−8.87	<.001	−.61
	Follow-up	3.12	0.82				

There were statistically significant (*P* < .001) reductions in low mood (PHQ-9), with average scores reducing from ‘moderate’ at session 1 to ‘mild’ at session 4 and follow-up. There were also statistically significant (*P* < .001) reductions in anxiety (GAD-7), with average scores reducing from ‘moderate’ at session 1 to ‘mild’ at session 4 and follow-up.

Furthermore, there statistically significant (*P* < .001) increases in resiliency (BRS), with average scores increasing from ‘low’ at session 1 to ‘normal’ at session 4 and follow-up. Finally, there were statistically significant (*P* < .001) increases in emotional wellbeing (WEMWBS), with scores increasing from ‘probable levels of depression’ at session 1 to ‘average mental wellbeing’ at session 4 and follow-up.

The GAD-7, PHQ-9 and WEMWBS all showed large effect sizes (*d* > 0.80), and the BRS showed a medium effect size (*d* > 0.60).

#### Qualitative feedback on patient benefit

The positive clinical impact of the T/APPs on patient outcomes was corroborated by feedback provided by patients, the T/APPs and General Practice staff. Patients reported feeling better after receiving support by a T/APP, while T/APPs and General Practice staff reported observing benefits to patients. See qualitative analysis below for full details (“Qualitative feedback”).

### Aim 2: the acceptability of the workforce and the role

#### Views on the T/APP role

Thirty-three (79%) General Practice staff members completed the online questionnaire about the T/APP role. This included 12 GPs, 6 practice managers, 4 team leaders, 4 social prescribers, 2 care coordinators, 2 MHPs, 1 nurse, 1 administrator and 1 ‘other’ staff member. Twenty-three (96%) T/APPs completed the online questionnaire about their role in the NHS. Finally, 240 patients completed the PEQ.

The results of the three questionnaires were anonymised, collated and analysed by an Assistant Psychologist trained in quantitative and qualitative data analysis. Data analysis was quality-checked by the Clinical Psychologist leading the service evaluation and a senior University Psychology lecturer.

##### Quantitative feedback

The results of the quantitative questions on the questionnaires were collated and summarised.

Thirty-two (97%) General Practice staff stated the addition of a T/APP practitioner had a positive impact on the service they worked in and the remaining 1 (3%) staff member stated they were unsure. Twenty-two (96%) T/APPs said they found that patients engaged with the support they offered and one said they were unsure. Finally, 18 (78%) T/APPs said they would recommend the T/APP role to other psychology graduates, 4 (17%) were unsure and 1 (4%) said no.

##### Qualitative feedback

An inductive summative content analysis was conducted on the qualitative feedback collected from General Practice staff, T/APPs and patients. Inductive analysis was selected as there is no previous research on this topic; therefore, categories and themes were extracted from the data rather than existing theory (Elo and Kyngäs, 2008). Furthermore, a summative approach was selected, meaning data were analysed through first coding and counting keywords, followed by the interpretation of underlying themes (Hsieh and Shannon, 2005).

**Figure 4.**
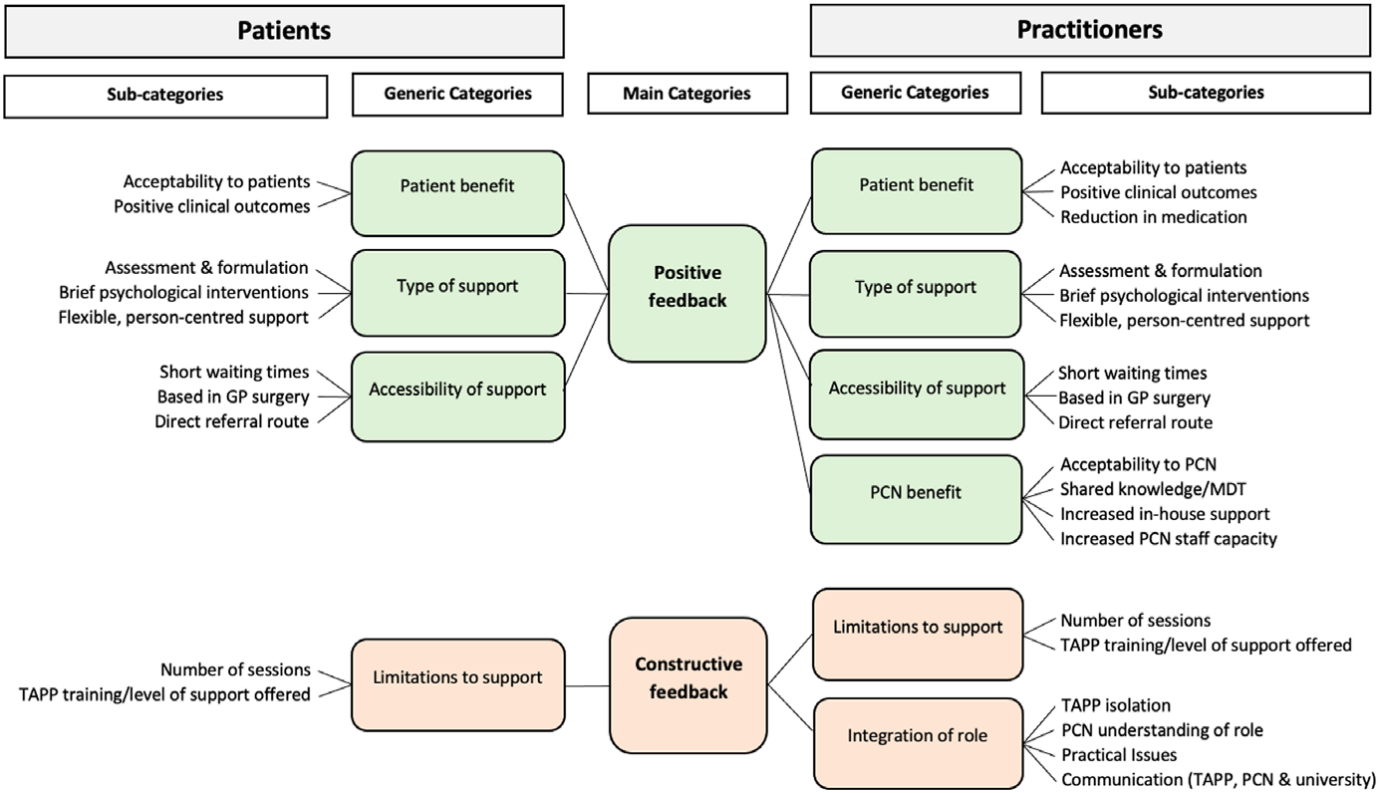
Main categories, generic categories and sub-categories found in content analysis of patients and practitioners (T/APPs and General Practice staff) view of the T/APP role.

In this service evaluation, firstly, an Assistant Psychologist assigned codes to words, phrases or sentences (utterances), individually across the three sources. The number of utterances assigned to each code were then counted (this data is available upon request). Second, an Assistant Psychologist and Clinical Psychologist made comparisons between the three groups, identifying significant overlap in the coding. This resulted in the decision to combine the data and present the results in terms of patient feedback and practitioner feedback (T/APPs and General Practice staff). Codes which were common to two or more sources were automatically included, while codes which were found in one source only remained in the analysis if they carried significant weight (e.g., a large number of utterances were recorded). Third, the coded, combined data were then analysed and sub-categories were derived. The sub-categories were then organised into more generic categories within two higher-level main categories: positive feedback and constructive feedback (see Figure 4).

The results of the content analysis revealed more positive sub-categories than critical sub-categories, reflective of a greater number of positively coded utterances. This indicates that the T/APP role was acceptable to patients, T/APPs and General Practice staff. Multi-source examples of utterances which make up the subcategories can be found in Table 3 (positive feedback) and Table 4 (constructive feedback).

**Table 3.** Examples of utterances relating to the main category of ‘positive feedback of the T/APP role’

Sub categories	Examples of utterances	
**Short waiting times**	PCN	*Great for patients who have received help quickly before their problems have escalated further*
	T/APP	Q*uick appointments (no waiting time)*
	Patient	*I thought it was good and I liked how quick I come off the waiting list, I didn’t expect that!*
**Based in GP surgery**	PCN	*Patients being able to see someone at the Surgery means that there is less stigma than seeing the Mental health team*
	T/APP	*Accessibility and normalisation of accessing mental health service in GP setting*
	Patient	*A service based in the GP surgery is useful as it is easy access and convenient for me to get to*
**Direct referral route**	PCN	*I have somewhere to signpost for immediate access to psychoeducation/brief therapy which is invaluable*
	T/APP	N/A
	Patient	*I feel lucky that I dropped into it when I did. That all came about by going to Dr’s for blood test and from that it evolved into this.*
**Assessment (understanding the problem)**	PCN	*Patients are seeing someone who can empathise and understand what they are going through*
	T/APP	*Assessment sessions to build rapport with patients and to understand their presenting problem.*
	Patient	*Yes – very helpful to help me to be clear on what the problem is that I need to solve*
**Brief psychological interventions (building skills)**	PCN	*New and additional clinical support to help local people access lower level, short psychological intervention*
	T/APP	*Brief interventions, e.g., behavioural activation, goal setting, coping strategies, mindfulness-based skills, thought challenging*
	Patient	*I’ve learnt lots of tips and strategies on how to manage my wellbeing*
**Flexible support (person-centred)**	PCN	*The T/APP was able to offer unique sessions which were tailored to our clients’ needs.*
	T/APP	*The varied, patient-centred approach and ability to offer a very different service to each patient as required*
	Patient	*The sessions felt organic and personal to me, rather than just following a checklist.*
**Acceptability to patients**	PCN	*Great feedback from patients*
	T/APP	*I have received a lot of positive patient feedback*
	Patient	Excellent, 10/10, 5 stars *****
**Positive clinical outcomes**	PCN	S*upported patients with early help and hopefully prevented further escalation of symptoms*
	T/APP	*This has been the best part; being able to see the improvement I have made supporting those who need mental health support*
	Patient	*Brilliant, I cannot thank you enough. I never thought I would be in this position 4 weeks from when I first spoke to you*
**Reduction in medication**	PCN	*There is no push to prescribe as we can try alternative methods for new mental health presentations first*
	T/APP	N/A
	Patient	N/A
**Acceptability to PCN**	PCN	Q*uick to integrate with the practice team, communicating the T/APP role to all staff*
	T/APP	*The surgery were very welcoming and accepting of my role*
	Patient	N/A
**Shared knowledge/MDT**	PCN	*The whole team has also benefitted from sharing good practice with, and learning from, our T/APP*
	T/APP	W*orking with GP staff to query symptoms of increased stress/anxiety/low mood/poor sleep earlier*
	Patient	N/A
**Increased in-house support**	PCN	*Our T/APP has been able to work with a cohort of patients that as a team we would have otherwise been unable to work with*
	T/APP	*It was great to be part of the broader push to enhance the service provision of primary care*
	Patient	N/A
**Increased PCN staff capacity**	PCN	*I have found the T/APP role very complimentary to my role – It has freed more of my appointments*
	T/APP	N/A
	Patient	N/A

**Table 4.** Examples of utterances relating to the main category of ‘constructive feedback of the T/APP role’

Sub-categories	Examples of utterances	
**T/APP isolation**	PCN	*N/A*
	T/APP	*Quite isolating being only psych practitioner in a service*
	Patient	*N/A*
**PCN understanding of role**	PCN	*At first it was a little bit confusing around what the service could offer*
	T/APP	*Have to be consistent, reminding them [clinical staff] of the service and who may be appropriate*
	Patient	*N/A*
**Practical issues**	PCN	*Space in our small surgery*
	T/APP	*Having to juggle a lot of admin with a heavy caseload*
	Patient	*N/A*
**Communication between T/APP, PCN and university**	PCN	*There needed to be a clearer communication stream between our own team, UCLAN and LSCFT*
	T/APP	*It did not seem that supervisors, managers and university colleagues communicated well with each other*
	Patient	*N/A*
**Number of sessions**	PCN	*Only being able to work with the patient for a limited time*
	T/APP	*4 sessions isn’t always enough, would like the flexibility to offer up to 6 for those who need it*
	Patient	*I wish it was more than 4 weeks*
**Training/level of support offered**	PCN	*Some of our patients have long established mental health or serious MH issues that they were not suitable for our T/APP*
	T/APP	*More training/more focus around needs that are perhaps more complex than the ‘prevention/promotion’ remit*
	Patient	*There were some things [the T/APP] isn’t able to do yet, would have liked to explore more CBT*

###### Patients

Patients responded positively to wellbeing support provided by the T/APPs. Almost all patients were both accepting of, and expressed benefiting from, the support they received. They reported liking the type of support provided, in terms of it being person-centred, helping them identify problem areas and build coping and wellbeing skills. Additionally, patients highlighted the benefits of the accessibility of the support, with it being in their local GP surgery and with shorter waiting times than other mental health services.

Constructive feedback provided by patients highlighted a desire for T/APPs to increase the number of wellbeing sessions to greater than 4. Additionally, some patients acknowledged limitations in the support the T/APP was able to provide (understandable given they are trainees) and suggested developing further skills may be of benefit.

###### Practitioners

The T/APP role was also received positively by practitioners (both General Practice staff and T/APPs). Their feedback mirrored that of patients, regarding 1) the benefits of the wellbeing sessions on patients, 2) the type of support the T/APP offered and 3) accessibility of the sessions. In addition to this, practitioners highlighted benefits of the T/APP role on the PCN. T/APPs expressed feeling like the support they offered was a valuable addition to the PCN. Meanwhile, General Practice staff highlighted that the T/APPs presence enabled them to learn more about mental health and wellbeing and allowed the PCN to widen and increase its clinical capacity.

Constructive feedback from practitioners echoed that of patients; they also suggested possible benefits to the T/APP offering more than four sessions and developing additional skills to widen the support offered. In addition to this, both T/APPs and General Practice staff identified challenges with integrating the T/APP role into the primary care setting. Some T/APPs expressed feeling isolated and not fully integrated into the General Practice team, even by the end of their training year. Furthermore, both T/APPs and PCNs acknowledged difficulties with General Practice staff understanding the function and remit of a T/APP and highlighted practical issues such as finding room space for the T/APP. Both T/APPs and General Practice staff suggested increased communication between the T/APP, the PCN and the university may be helpful to resolve these issues.

## Discussion

### Summary

The new roles (ARRs) coming into PCN settings provide an opportunity to help existing clinicians meet the demand for mental health care. General Practice is the perfect setting for timely or early intervention to prevent deterioration. This important and novel service evaluation explored the efficacy of brief psychological interventions, delivered within General Practice settings, and found both clinically and statistically significant reductions on measures of anxiety and depression and clinical and statistically significant improvements on measures of resilience and wellbeing. Multi-source feedback from individuals who accessed the service, the workforce and General Practice staff was very positive, with benefits for patient care, a positive impact for other practice staff and suitability of service location all cited as benefits. The main challenge related to developing new roles in this setting and service integration.

### Strengths and limitations

This service evaluation captured pre- and post-clinical outcome data for over 500 people, half of whom also then completed follow-up metrics. The data were collated from over half of all PCNs across the Lancashire and South Cumbria footprint. There was a good response rate for the qualitative questionnaires. From this information, strong conclusions about the positive impact the T/APPs have had, and are having, can be drawn.

In addition to the patients who engaged with all five sessions, there was also clinical benefit to the assessment-only sessions offered by the T/APPs. In comparison to a brief 10-min appointment with a GP, a 45-min assessment session allowed patients longer to tell their story, advice and guidance to be given and the right referral to the right service to be made.

It was an aim of the T/APP provision to offer a service that mirrored that of General Practice, an all-age service. The demographics suggest that this was successfully achieved. However, the data collated in relation to gender and ethnicity suggest that more needs to be done in terms of engaging with males and those from ethnic minority groups. For the second year of service evaluation, a working party will be set up with a clear directive to understand these engagement figures and set actions for improvement.

Whilst, at least in theory, anyone could benefit from psychological work focusing on promoting positive emotional wellbeing and preventing deteriorating, as the T/APPs were limited to providing brief interventions, their focus was aimed at those who would be classed as ‘pre-caseness’ or having mild difficulties. However, many individuals presented with ‘moderate’ difficulties, according to the psychometric scores. Although an initial referral was perhaps for ‘work stress’ (for example), by the end of the assessment session, for some, it became apparent that the individual would be accepted for intervention within IAPT (adult primary care mental health) services. However, often the client may decline this referral due to a preference to be seen within their local GP surgery, or a wish to try working with the T/APP first, then re-consider a referral once the sessions with the T/APP had ended. In this way, many clients benefitted sufficiently from a brief intervention that they did not then request or require on ongoing referral to, for example, IAPT. Further investigation about the cost-effectiveness of this balance would be beneficial.

A difficulty highlighted by T/APPs and General Practice staff was the integration of the T/APPs into PCNs. This challenge was anticipated, given it was the first year of the T/APP deployment and psychology roles within General Practice settings are unusual. Integration is anticipated to become easier as the T/APP course develops, and the role becomes more widely known about.

### Comparison with existing literature

Whilst there has been work done in the area of mental health prevention and promotion (for a recent review, see Budd *et al.*, 2021), this is still an area lacking in research. The current efficacy evaluation helps to strengthen the argument of the importance of such a focus in General Practice settings.

The King’s Fund (Baird and Beech, 2020) highlighted that collaboration in primary care can take time and strong relationships, a shared vision and effective leadership are all crucial. This was clear in the qualitative feedback from the workforce and PCN staff members that the main cited challenge was integration and developing a new role. Successful integration relies on effective and healthy working relationships, which of course take time to build. Whilst associated social distancing restrictions as a result of the COVID-19 pandemic presented challenges, the move to communicating through virtual means, such as MS teams, also enhanced the ability to communicate with numerous practices, over a wide geographical location in the same working day. There were challenges relating to establishing a new role and ensuring all practice staff members understood the role and service provided, particularly when this workforce can be transient (e.g., locum roles).

The T/APPs offer a genuine and sustainable workforce solution for mental health services. Workforce supply issues are a known concern (Beech *et al.*, 2019; British Medical Journal, 2021), and the creation of new roles to meet the growing demand is a good opportunity. This fits well with recommendations (NHS England, 2016; Thomas *et al.*, 2016; Mind, 2018) focusing upon having a wider range of practice staff within primary care settings who can support mental health care.

### Implications for research and/or practice

To strengthen this evaluation further, further research is needed. In particular, a comparison against a control group would be beneficial. Feedback from patients was often about providing more sessions; however, it is currently unclear if that would bring more clinical benefit or not.

From a research perspective, it will also be important to follow-up patients over a longer time period to understand if the statistically and clinically significant positive changes in measures are maintained. In addition, our evaluation of this service would be strengthened if conclusions about cost-effectiveness or economic impact of the service could be included. For the second cohort of T/APPs, it is recommended that both the EuroQoL-5 Dimensions-5 Levels (EQ-5D-5L) questionnaire be administered during the first, fourth and follow-up appointments and a patients’ resource-user questionnaire to estimate the number of and change in patients’ consultations, medication, therapies and further referrals be evaluated.

The current service delivery model is being reviewed and will be altered in response to the feedback received after this first pilot year. An ‘assess and refer’ service has been trialled, and a larger trial will now be rolled out. This relates to when a qualified APP conducts 30-min triage appointments with patients who are booked in straight from reception or online. This of course will directly reduce the demand upon GP and nurse time. This ‘assess and refer’ aspect to the T/APP role is different to the brief interventions they can provide. It worked well when T/APPs completed assessments of people presenting with mental health difficulties and then referring on to the most appropriate service as need required.

There are immeasurable benefits to the new T/APP workforce; in 2022, there will be 17 qualified APPs working in Lancashire and South Cumbria and 17 more new T/APPs joining PCN teams. T/APPs are a good workforce supply for the ARRs roles, but it takes time to develop a workforce. Development of the Band 5 APP role and structured career pathway and progression are priority areas, so the PCNs can continue to benefit from the added value of this new sustainable workforce.

## Conclusion

The overall objective of this service evaluation has been achieved; the T/APPs clearly add value to mental health care in General Practice settings. The aims to understand the clinical impact and efficacy of the mental health prevention and promotion service and the acceptability of the T/APP role were met. The service the T/APPs delivered was both clinically effective and acceptable to patients, General Practice staff and psychology graduates.

There are some larger implications of this evaluation to consider. The T/APPs are a genuinely new workforce supply of MHPs who can deliver brief psychological interventions in primary care settings to help meet the ambitions of the NHS LTP. With the new ARRs MHP roles joining PCN settings, the T/APPs provide a workforce solution to help meet the demand for mental health care and MHPs within this setting. There is potential for this workforce initiative to spread outside of the North West of England, so evaluating impact and understanding added value is crucial. Local Workforce Modelling (using the Workforce Repository and Planning Tool – WRaPT) has demonstrated that the T/APPs have the potential to free up GP capacity, which, given the highlighted reduced GP workforce (e.g., NHS Digital, 2022), is an important point. This evaluation has demonstrated that the T/APPs bring clinical and service benefit by providing the assessment-only appointments, and a further area for research would be to evaluate the impact seeing a T/APP for a brief intervention would have on future health care use. In addition, further evaluation will also include an economic appraisal and a longer-term follow-up period. It will also be important to support the T/APPs develop in their role and understand how to support these practitioners to develop their career, their career pathway and ensure primary care can retain this valuable workforce.

## References

[ref28] Alem A , Jacobsson L and Hanlon C (2008) Community-Based mental health care in Africa: mental health workers’ views. World Psychiatry 7, 54. 18458779 10.1002/j.2051-5545.2008.tb00153.x PMC2327237

[ref1] Baird B and Beech J (2020) Primary care networks explained. London, UK: The Kings Fund. Retrieved May 2022 from https://www.kingsfund.org.uk/publications/primary-care-networks-explained.

[ref30] Bassilios B , Pirkis J , Fletcher J , Burgess P , Gurrin L , King K , Kohn F and Blashki G (2010) The complementarity of two major Australian primary mental health care initiatives. Australian & New Zealand Journal of Psychiatry 44, 997–1004. 21034182 10.3109/00048674.2010.495052

[ref2] Beech J , Bottery S , Charlesworth H , Gershlick B , Hemmings N , Imison C , Kahtan P , McKenna H , Murray R and Palmer B (2019) Closing the gap. Key areas for action on the health and care workforce. London, UK: The Health Foundation. The Kings Fund. Nuffield Trust. Retrieved May 2022 from https://www.kingsfund.org.uk/sites/default/files/2019-03/closing-the-gap-health-care-workforce-overview_0.pdf.

[ref3] Bowen D , Kreuter M , Spring B , Cofta-Woerpel L , Linnan L , Weiner D , Bakken S , Kaplan C , Squiers L , Fabrizio C and Fernandez M (2009) How we design feasibility studies. American Journal of Preventive Medicine 36, 452–457. 19362699 10.1016/j.amepre.2009.02.002 PMC2859314

[ref4] British Medical Journal (2021) Supporting General Practice in 2021/22. Retrieved 06 January 2022 from bma-c1054-supporting-general-practice-in-202122-21-january-2021.pdf.

[ref5] Budd M , Iqbal A , Harding C , Rees E and Bhutani G (2021) Mental health promotion and prevention in primary care: What should we be doing vs. what are we actually doing? Journal of Mental Health and Prevention 21.

[ref6] Cameron IM , Crawford JR , Lawton K and Reid IC (2008) Psychometric comparison of PHQ-9 and HADS for measuring depression severity in primary care. British Journal of General Practice 58, 32–36. 10.3399/bjgp08X263794 PMC2148236 18186994

[ref7] Carbone SR (2020) Flattening the curve of mental ill-health: the importance of primary prevention in managing the mental health impacts of COVID-19. Mental Health & Prevention 19, 200185. 10.1016/j.mhp.2020.200185. 32566473 PMC7255235

[ref8] Department for Health and Social Care (2017) A framework for mental health research over the next decade. Retrieved May 2022 from https://www.gov.uk/government/publications/a-framework-for-mental-health-research.

[ref9] Elo S and Kyngäs H (2008) The qualitative content analysis process. Journal of Advanced Nursing 62, 107–115. 18352969 10.1111/j.1365-2648.2007.04569.x

[ref10] Health Education England (2017) Stepping forwards to 2020/21: the mental health workforce plan for England. London, UK: HEE. Retrieved May 2022 from https://www.hee.nhs.uk/sites/default/files/documents/Stepping%20forward%20to%20202021%20-%20The%20mental%20health%20workforce%20plan%20for%20england.pdf.

[ref11] Hsieh H and Shannon S (2005) Three approaches to qualitative content analysis. Qualitative Health Research 15, 1277–1288. 16204405 10.1177/1049732305276687

[ref12] Jia R , Ayling K , Chalder T , Massey A , Broadbent E , Coupland C and Vedhara K (2020) Mental health in the UK during the COVID-19 pandemic: cross-sectional analyses from a community cohort study. British Medical Journal Open 10. 10.1136/bmjopen-2020-040620 PMC7493070 32933965

[ref13] Knapp M and McDaid D (2011) Mental health promotion and mental illness prevention: the economic case. London: Department of Health.

[ref14] Kroenke K , Spitzer R and Williams J (2001) The PHQ-9: validity of a brief depression severity measure. Journal of General Internal Medicine 16, 606–613. doi: 10.1046/j.1525-1497.2001.016009606.x. 11556941 PMC1495268

[ref29] Lockhart C (2006) Collaboration and referral practices of general practitioners and community mental health workers in rural and remote Australia. Australian Journal of Rural Health 14, 29–32. 16426430 10.1111/j.1440-1584.2006.00746.x

[ref15] London Strategic Clinical Network for Mental Health (2014). A commissioner’s guide to primary care mental health. London, UK: London Strategic Clinical Network for Mental Health. Retrieved May 2022 from https://mentalhealthpartnerships.com/resource/a-commissioners-guide-to-primary-care-mental-health/.

[ref16] Mavali S , Mahmoodi H , Sarbakhsh P and Shaghaghi A (2020) Psychometric properties of the Warwick–Edinburgh Mental Wellbeing Scale (WEMWBS) in the Iranian older adults. Psychological Research & Behaviour Management 13, 693–700. doi: 10.2147/PRBM.S256323. PMC7443441 32884372

[ref17] Mind (2018) ‘40% of all GP appointments about mental health’. *Mind website*. Retrieved 19 August 2020 from www.mind.org.uk/news-campaigns/news/40-per-cent-of-all-gp-appointments-about-mental-health.

[ref18] Molodynski A , McLellan A , Craig T and Bhugra D (2021) What does COVID mean for UK mental health care? International Journal of Social Psychiatry 67, 823–825. 32517530 10.1177/0020764020932592 PMC8559169

[ref19] Naylor C , Parsonage M , McDaid D , Knapp M , Fossey M and Galea A (2012) Long-term conditions and mental health: the cost of co-morbidities. London, UK: The King’s Fund and the Centre for Mental Health. Retrieved May 2022 from https://www.kingsfund.org.uk/sites/default/files/field/field_publication_file/long-term-conditions-mental-health-cost-comorbidities-naylor-feb12.pdf.

[ref20] NHS Digital (2022) General practice workforce. Retrieved 07 August 2022 from https://digital.nhs.uk/data-and-information/publications/statistical/general-and-personal-medical-services/30-june-2022.

[ref21] NHS England (2016) General practice forward view. Retrieved 01 November 2021 from https://www.england.nhs.uk/wp-content/uploads/2016/04/gpfv.pdf.

[ref22] NHS England (2019) NHS long term plan. NHS England, 2019. Retrieved from http://www.england.nhs.uk/long-term-plan.

[ref23] Public Health England (2020) Prevention concordant for better mental health. Retrieved May 2022 from https://www.gov.uk/government/publications/prevention-concordat-for-better-mental-health-consensus-statement/prevention-concordat-for-better-mental-health.

[ref24] Smith B , Dalen J , Wiggins K , Tooley E , Christopher P and Bernard J (2008) The brief resilience scale: assessing the ability to bounce back. International Journal of Behavioral Medicine 15, 194–200. 18696313 10.1080/10705500802222972

[ref25] Spitzer R , Kroenke K , Williams J , and Lowe B (2006) A brief measure for assessing generalized anxiety disorder. Archives of Internal Medicine 166, 1092–1097. doi: 10.1001/archinte.166.10.1092. 16717171

[ref26] Tennant R , Hiller L , Fishwick R , Platt S , Joseph S , Weich S and Stewart-Brown S (2007) The Warwick-Edinburgh mental well-being scale (WEMWBS): development and UK validation. Health and Quality of Life Outcomes 5, 63. doi: 10.1186/1477-7525-5-63. 18042300 PMC2222612

[ref27] Thomas S , Jenkins R , Burch T , Calamos Nasir L , Fisher B , Giotaki G , Gnani S , Hertel L , Marks M , Mathers N , Millington-Sanders C , Morris D , Ruprah-Shah B , Stange K , Thomas P , White R and Wright F (2016) Promoting mental health and preventing mental illness in general practice. London Journal Primary Care (Abingdon) 24, 3–9. 10.1080/17571472.2015.1135659 PMC5330334 28250821

[ref31] WHO Europe (2008) Policies and practices for mental health in Europe – meeting the challenges. www.euro.who.int/en/publications/abstracts/policies-andpractices-for-mental-health-in-europe.-meeting-the-challenges.

